# Molecular Diversity and Distribution of Arbuscular Mycorrhizal Fungi at Different Elevations in Mt. Taibai of Qinling Mountain

**DOI:** 10.3389/fmicb.2021.609386

**Published:** 2021-03-04

**Authors:** Mengge Zhang, Zhaoyong Shi, Mei Yang, Shichuan Lu, Libing Cao, Xugang Wang

**Affiliations:** ^1^College of Agriculture, Henan University of Science and Technology, Luoyang, China; ^2^Luoyang Key Laboratory of Symbiotic Microorganism and Green Development, Luoyang, China; ^3^Henan Engineering Research Center of Human Settlements, Luoyang, China

**Keywords:** arbuscular mycorrhizal fungi (AMF), molecular diversity, community composition, elevational gradient, mountain ecosystem

## Abstract

Arbuscular mycorrhizal fungi (AMFs) play a vital role in ecosystems, especially in ecosystem variability, diversity, and function. Understanding the AMF diversity, distribution, and their driver at different altitudinal gradients is a benefit for understanding the ecological function of AMF in mountain ecosystems. In this study, we explored the AMF molecular diversity and their distribution from 660 to 3,500 m a.s.l. in Mount Taibai of Qinling Mountains based on high-throughput sequencing technology. A total of 702 operational taxonomic units (OTUs) in 103 species of AMF are isolated from soil samples, which belong to 18 identified and 1 unidentified genus in 10 families. The fungi in the genus of *Glomus* is the most dominant, with the occurrence frequency of 100% and the relative abundance of 42.268% and 33.048% on the species and OTU level, respectively. The AMF colonization in root could be simulated by a cubic function with the change of altitudes with the peak and trough at a.s.l. 1,170 and 2,850 m, respectively. Further, AMF diversity indices including Sob, Shannon diversity, and Pielou evenness also showed the same cubic function change trends with increasing altitude at OTU and species levels. However, the average values of diversity indices at OTU level are always higher than these at the species level. Based on the OTU level, the highest and lowest values of Shannon and Pielou indices are observed at the altitudes of 1,400 and 2,800 m, respectively. The pattern of AMF community distribution in Mt. Taibai is driven by altitude with the characteristics of more abundance in the medium- to low-altitude than high-altitude areas. In general, abundant AMF molecular diversity and species exit in different elevations of Mt. Taibai, which indicate gradient changes with elevations.

## Introduction

Arbuscular mycorrhiza (AM) is an obligate symbiotic formed between arbuscular mycorrhizal fungi (AMFs) and plant roots. As one of the richest soil symbiotic microorganisms, AMFs are associated with 80% terrestrial plant species (Smith and Read, 2008), which play important roles in regulating the relationship between soil and plants during nutrient cycling in the ecosystem, such as C (Bi et al., 2020; Zhou et al., 2020), N (Tedersoo et al., 2020), P (Liu et al., 2018; Sanders and Rodriguez, 2016), and so on. Studies have shown that AMF plays an important role in regulating plant species diversity and the ecosystem succession (van der Heijden and Scheublin, 2007; Jing et al., 2015; Bauer et al., 2017). Therefore, exploring AMF diversity in different ecosystems has become a research hotspot (van der Heijden et al., 1998; Hontoria et al., 2019).

AMF diversity has been reported in various ecosystems, such as grassland ecosystem (Liang et al., 2017; Smilauer et al., 2019; Goldmann et al., 2020), agricultural ecosystem (Oehl et al., 2010; Liu et al., 2019; Zhu et al., 2020), forest ecosystem (Dos Passos et al., 2020; Rozek et al., 2020), and even global ecosystem (Tedersoo et al., 2014; Davison et al., 2015). Because of the strong environmental gradient, mountain ecosystem provides a unique opportunity for the study of AMF diversity and becomes a key area for global biodiversity research (Zanzottera et al., 2020). In mountain ecosystems, elevation gradient causes environmental gradient changes, which may have a greater impact on AMF community and diversity. Studies have shown that it is usually observed that as the altitude increases, the diversity of AMF presents a decreasing trend in temperate climate (Liu et al., 2015; Vieira et al., 2019). However, this pattern is not constant, and Coutinho et al. (2015) found an increased trend of AMF diversity at high altitudes in the tropical ecosystem of Brazil.

The exploration of AMF diversity in different ecosystems by molecular biological technology is becoming an increasingly popular method, which not only enriches and improves the classification system of AMF but also enables us to accurately evaluate the AMF diversity and community distribution. However, AMF diversity mainly depends on morphological characteristics of spores isolated from soils at numerous previous reports (da Silva et al., 2014; Sale et al., 2015; Melo et al., 2020). It is tempting to ask the question of whether AMF diversity is consistent between molecular and traditional morphological approaches. This seems a similar conclusion that more AMF taxa are always identified by molecular than traditional morphological methods (Senés-Guerrero and SchuSSler, 2016; Silvani et al., 2017). Kivlin et al. (2011) and Opik et al. (2014) combined all reported AMFs in the global ecosystem and found that the number of molecular taxa with 350 to 1,000 is much higher than that of morphological species with about 250. Bidondo et al. (2018) detected AMFs associated with pecan trees and showed that AMF richness identified by molecular was between three and five times higher than estimated by morphological methods. The development of molecular biology techniques allows detection of closely related species and enables their response to regional environmental gradients to be recorded (Chamkhi et al., 2018). Mountain ecosystem has complex structures and functions due to their pronounced climatic gradients, which is of great significance for the study of biodiversity (Zanzottera et al., 2020; Zhang and Liu, 2011). Further, research has shown that understanding AMF diversity and community distribution in the mountains was the basis for predicting the occurrence and evolution of symbiosis in mountain ecosystems (Li et al., 2018).

The Qinling Mountains provide an ecological transition zone between the warm temperate and the subtropical and are one of the richest species regions in China (Shi et al., 2014; Shuai et al., 2017). As the main peak of the Qinling Mountains, the Mount Taibai (Mt. Taibai) has an obvious vertical climate spectrum and a complex vertical vegetation spectrum, which provides more possibilities for the biodiversity in this study.

Shi et al. (2014) studied AMF diversity and identified 63 AMF belonging to 12 genera in Mt. Taibai with the morphological taxonomy. In this study, we researched the AMF molecular diversity and distribution mechanism by molecular biology methods in Mt. Taibai of the Qinling mountains, with the following objectives for determining the changes of AMF molecular diversity and community distribution on altitudinal gradients in mountain ecosystems and testing the differences of AMF diversity and their distribution with elevations based on between molecular and morphological approach. Furthermore, we hope to enrich the ecological theory of AMF diversity by providing supporting data by varying elevations in mountain ecosystem. On the other hand, we hope to explore ecological service functions of AMF in mountain ecosystem and provide a theoretical basis for the response of AMF to climate change.

## Materials and Methods

### Study Region

This study was carried out in Mt. Taibai (33°49′31″–34°08′11″ N, 107°41′23″–107°51′40″ E). It is located in Shaanxi province and is the highest mountain in the east of China’s mainland, with an elevation of 3,767.2 m. It ranges 61 km from east to west and 39 km from north to south. The mean annual temperature is 8°C, and the average annual precipitation ranges from 750 to 950 mm. The vertical effect on Mt. Taibai climate is obvious. With the elevation rise, it can be successively divided into the warm temperate zone (<1,300 m), cold temperate zone (1,500–3,000 m), subfrigid zone (3,000–3,350 m), and frigid zone (>3,350 m). And the annual average temperatures in different climate zones are 11°C, 6°C, −1°C, and less than 8°C, respectively (Baidupedia Contributors, 2020).

Because of the special geographical location and large elevation gradients, the climate, terrain, and soil in the territory are complex and diverse. The flora is also particularly rich, and it is one of the most abundant areas of temperate flora in China (Zhang and Liu, 2011; Yao et al., 2020). According to the survey, there were more than 1,700 seed-bearing plants found there, which account for a large proportion of the flora in Qinling mountain. In the investigation of endemic plants in Mt. Taibai, it was found that perennial herbs were the most common, accounting for 61% of the total endemic plants (Baidupedia Contributors, 2020). Besides, the soil types of Mt. Taibai include marsh soil, cinnamon soil, brown earth, alpine meadow soil, and so on. Complex and diverse soil types provide more possibilities for Mt. Taibai biodiversity.

### Collection of Samples

Soil samples were collected from 660 to 3,500 m at 12 elevation gradients including 660, 1,170, 1,400, 1,800, 2,100, 2,200, 2,500, 2,650, 2,850, 3,100, 3,250, and 3,500 m in Mt. Taibai. At every target altitude, three 10 × 10 m quadrats were set up, with a distance of more than 50 m from each other. Each quadrat was divided into 25 subplots with sides of 2 × 2 m. Four soil cores with a diameter of 5 cm and a depth of 0–30 cm were collected randomly in each subplot. Then, for each quadrat, a total of 100 soil cores were gathered into a mixed sample. Soil mixed subsamples and corresponding root segments (2–5 g) were refrigerated in a cooling bin and taken immediately to the laboratory to assess AMF diversity and root colonization. AMF operational taxonomic units (OTUs) were clustered with 97% similarity cutoff using UCLUST.

### Identification of AMF

Genomic DNA was extracted from soil samples using E.Z.N.A. soil DNA Kit (Omega Bio-tek, Norcross, GA, United States) following the manufacturer’s protocols. The quality of extracted DNA was checked by 1% agarose gel electrophoresis and spectrophotometer at a ratio of 260 nm. All extracted DNA samples were stored at -20°C for further analysis. The extracted DNA was amplified using a polymerase chain reaction (PCR) procedure. The AMF region was amplified on Eppendorf Mastercycler Gradient Thermocycler (Germany), with the universal primers FLR3-F (5- TTGAAAGGGAAACGATTGAAGT-3) and FLR4-R (5- TACGTCAACATCCTTAACGAA-3). The 5′ ends of the both primers were tagged. The ultra PAGE purified primers were ordered from Invitrogen, China. The PCR mixtures were as follows: 12.5 μL KAPA 2G Robust Hot Start Ready Mix, 1 μL forward primer (5 μM), 1 μL reverse primer (5 μM), 5 μL DNA (total template quantity is 30 ng), and 5.5 μL H_2_O. Thermocycling consisted of an initial denaturation at 95°C for 5 min, followed by 28 cycles of 95°C for 45 s, 55°C for 50 s, 72°C for 45 s, and a final extension at 72°C for 10 min. Three separate reactions were conducted to account for potentially heterogeneous amplification from the environmental template for each sample. PCR products were extracted from 2% agarose gels and purified using the AxyPrep DNA Gel Extraction Kit (Axygen Biosciences, Union City, CA, United States) then quantified using QuantiFluor -ST (Promega, United States). An equimolar mix of all three amplicon libraries was used for sequencing at Allwegene Company, China. Raw sequence data for all the samples were uploaded to the NCBI Sequence Read Archive under accession number PRJNA692777.

### Bioinformatics Analysis of Sequence Data

The extraction of high-quality sequences was first performed with the QIIME package (Quantitative Insights Into Microbial Ecology) (v1.2.1). Raw sequences were selected based on sequence length, quality, primer, and tag. The raw sequences were selected, and the low-quality sequences were removed. The quality filtering used the following criteria: (i) raw reads were shorter than 110 nucleotides; (ii) the 300-bp reads were truncated at any site receiving an average quality score < 20 over a 50-bp sliding window, discarding the truncated reads that were shorter than 50 bp; (iii) exact barcode matching, two-nucleotide mismatch in primer matching, and reads containing ambiguous characters were removed; (iv) only sequences that overlap longer than 10 bp were assembled according to their overlap sequence. Reads that could not be assembled were discarded.

The unique sequence set was classified into OTUs under the threshold of 97% identity using UCLUST (Caporaso et al., 2012). Chimeric sequences were identified and removed using Usearch (version 8.0.1623). The taxonomy of rRNA gene sequence was analyzed by UCLUST against the Silva database using a confidence threshold of 90%.

### AM Colonization and Soil Properties Analysis

Fresh fine roots were washed and put them in a water bath of 10% (m/v) KOH at 90°C for 20–30 min. When the roots are relatively transparent, the dye is rinsed off, and 5% acetic acid was added to soak for 5 min, and then they were dyed with 5% acetic acid ink. The root samples were cut into1.0-cm-long segments. Then they were mounted on glass slides dripped with polyvinyl alcohol solution, and the presence of the AMF structure was checked under the Motic BA310 microscope at 100–400 times magnification. According to the number of mycorrhizal structures in each segment of root system, 0%, 10%, 20%, 30%… 100%, the infection rate of each root segment was determined. The mycorrhizal colonization can be calculated according to the formula: Σ (0 × the number of root segments + 10% × the number of root segments + 20% × the number of root segments + … + 100% × the number of root segments)/observed total number of root segments.

The concentrations of soil C and N were determined by element analyzer instrument. And the concentration of soil available phosphorus was determined using Olsen and Sommers (1982) method. Soil pH was determined on a 1:2.5 suspension of soil in water using a pHS-3D digital pH meter.

### Calculations of AMF Molecular Diversity

Species richness, frequency, and relative abundance of AMF were determined as follows:

(1) The occurrence frequency of a specified AMF genus was defined as the percentage of the number of samples in which the genus was observed to the total number of samples.

(2) The relative abundance of a genus based on species or OTU level was calculated as the percentage of the number of species or OTUs for each genus divided by total numbers of species or OTUs in all genera.

(3) Shannon–Weiner index: the *P*_*i*_ of AM fungal species or OTUs was defined as the percentage of the sequences for each species or OTUs detected to total species or OTU sequences in a sample.

***H* = −Σ [*P*_*i*_log_2_ (*P*_*i*_)]**

(4) The Sobs index of AM fungal species or OTU was defined as the numbers of species or OTUs in a sample.

(5) The Pielou index:

***E* = *H*/*H*_*max.*_*H*_*max.*_ = ln (sobs)**

### Statistical Data Analysis

The results of root colonization and AMF diversity index were presented by arithmetic means. The mycorrhizal colonization was analyzed by one-way analysis of variance at the *P* < 0.05 level among 12 altitudes, and the Duncan multiple-comparisons method was used to test the significance of the difference. The unique sequence set was classified into OTUs under the threshold of 97% identity using UCLUST, to generate rarefaction curves and to calculate the richness and diversity indices. We used the Sobs index, Shannon index, and Pielou index to estimate the richness, alpha diversity, and evenness of the AMF on species and OTU levels. In order to investigate the relationships between AMF diversity indices and elevation gradient, a curve regression analysis was conducted, and the explained variability (*R*^2^) was also estimated. In addition, we used the Pearson correlation analysis to evaluate the significance of AMF diversity and soil factors. The statistical tests were carried out in SPSS software 21.0.

## Results

### Changes of Arbuscular Mycorrhizal Colonization in Roots With Elevations

**FIGURE 1:**
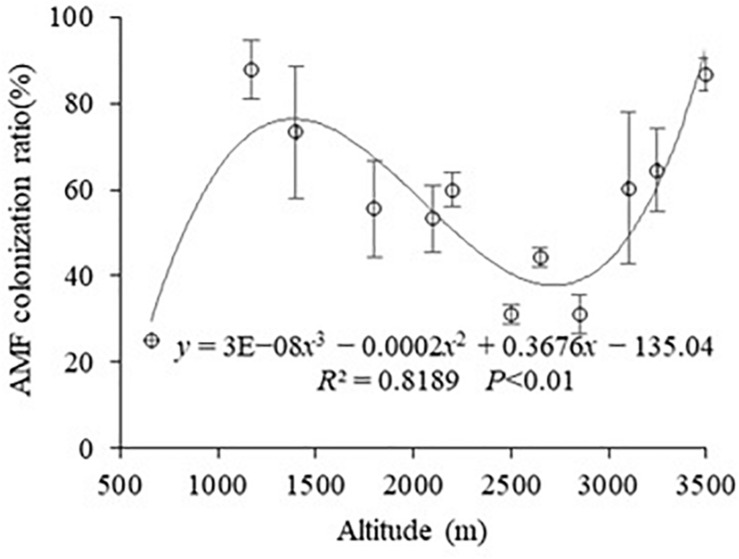
Change in percentage of AM colonization among different altitudes.

It was found that all samples were colonized by AMF by measuring the root colonization at different altitudes in Mt. Taibai (Figure 1). The colonization of AM varied from 25% to 87.71%, with an average 56.1%. The change trend of root colonization tended to a form of cubic function with the *R*^2^ was 81.89%, and *P* was less than 0.001 with the change of altitudes. The mycorrhizal colonization increased between 660 and 1,170 m and then gradually declined between 1,170 and 2,850 m, followed by a further increment between 2,850 and 3,500 m. The highest colonization occurred at 1,170 m, and the lowest appeared at 660 m, which is almost in line with the mathematical model of the cubic function.

### Community Composition and Distribution of AMF in Different Elevations

A total of 702 OTUs belonged to 103 AMF species, which were subordinate to 18 identified and 1 unidentified genus representing 10 families (Table 1, Supplementary File 2). The unidentified genus only occurred at the altitude of greater than 2,500 m. Eight of these genera appeared at all altitudes including *Acaulospora*, *Ambispora*, *Claroideoglomus*, *Funneliformis*, *Glomus*, *Dominikia*, *Rhizophagus*, and *Septoglomus*. In addition, *Corymbiglomus* was only identified at 1,170 m.

**TABLE 1:** The distribution of AMF family, genus, and the number of AMF species and OTUs among different altitudes in soil samples collected from Mt. Taibai.

**Family**	**Genus**	**Altitude (m)**												**Total**
		**660**	**1,170**	**1,400**	**1,800**	**2,100**	**2,200**	**2,500**	**2,650**	**2,800**	**3,100**	**3,250**	**3,500**	
*Acaulosporaceae*	*Acaulospora*	8 (10)	2 (2)	4 (5)	6 (9)	6 (37)	7 (12)	5 (33)	4 (22)	10 (25)	9 (39)	6 (20)	5 (18)	13 (81)
	*unidentified*	0	0	0	0	0	0	1 (3)	1 (5)	1 (11)	0	1 (3)	0	1 (11)
*Ambisporaceae*	*Ambispora*	1 (3)	1 (4)	1 (5)	1 (4)	1 (4)	1 (6)	1 (7)	1 (9)	1 (9)	1 (1)	1 (9)	1 (3)	1 (14)
*Claroideoglomeraceae*	*Claroideoglomus*	2 (6)	2 (5)	3 (7)	2 (6)	2 (5)	1 (6)	3 (4)	3 (5)	2 (3)	1 (2)	1 (1)	2 (2)	4 (17)
*Diversisporaceae*	*Corymbiglomus*	0	1 (1)	0	0	0	0	0	0	0	0	0	0	1 (1)
	*Diversispora*	3 (4)	1 (1)	1 (1)	0	5 (11)	3 (5)	4 (5)	2 (2)	1 (1)	2 (6)	0	2 (2)	5 (17)
	*Redeckera*	0	0	0	0	1 (1)	1 (1)	1 (2)	0	0	1 (1)	0	0	1 (2)
*Entrophosporaceae*	*Entrophospora*	0	1 (1)	1 (1)	0	2 (2)	1 (1)	0	0	0	0	0	0	2 (2)
*Gigasporaceae*	*Scutellospora*	1 (1)	1 (2)	1 (1)	0	0	0	2 (2)	2 (2)	2 (2)	2 (2)	1 (1)	1 (1)	2 (3)
*Glomeraceae*	*Funneliformis*	2 (5)	2 (2)	2 (2)	1 (1)	2 (2)	3 (3)	2 (8)	2 (9)	2 (5)	2 (2)	2 (4)	2 (7)	3 (12)
	*Glomus*	20 (53)	31 (117)	26 (88)	28 (137)	26 (61)	25 (76)	24 (69)	18 (34)	14 (33)	10 (22)	22 (49)	19 (49)	44 (232)
	*Kamienskia*	2 (4)	2 (20)	2 (15)	2 (18)	1 (4)	2 (8)	0	0	1 (2)	0	2 (4)	1 (1)	2 (27)
	*Dominikia*	1 (1)	2 (3)	1 (1)	2 (2)	1 (1)	1 (1)	1 (1)	1 (1)	1 (1)	1 (2)	1 (1)	1 (1)	3 (5)
	*Rhizophagus*	3 (11)	4 (19)	4 (11)	4 (18)	3 (16)	4 (15)	2 (2)	3 (8)	3 (3)	2 (2)	3 (11)	4 (22)	9 (50)
	*Sclerocystis*	1 (2)	0	0	0	0	0	1 (2)	0	0	0	1 (1)	1 (2)	1 (2)
	*Septoglomus*	4 (79)	7 (133)	5 (127)	6 (147)	6 (128)	8 (138)	6 (118)	4 (112)	4 (53)	4 (42)	3 (41)	4 (92)	8 (218)
*Pacisporaceae*	*Pacispora*	1 (1)	0	0	0	0	0	1 (2)	0	0	1 (5)	1 (1)	1 (1)	1 (5)
*Paraglomeraceae*	*Paraglomus*	0	0	0	0	1 (1)	1 (1)	0	0	1 (1)	0	1 (1)	0	1 (1)
*Sacculosporaceae*	*Sacculospora*	1 (1)	0	0	0	1 (1)	0	1 (2)	1 (2)	1 (2)	1 (2)	0	0	1 (2)
Total		50 (181)	57 (310)	51 (264)	52 (342)	58 (274)	58 (273)	55 (260)	42 (211)	44 (151)	37 (128)	46 (147)	44 (201)	103 (702)

Of the 103 species, 43 species belonged to the genus of *Glomus*, which accounted for the total percentage of 44.3%. However, only one species was identified in the genera of *Ambispora*, *Corymbiglomus*, *Redeckera*, *Sclerocystis*, *Pacispora*, *Paraglomus*, and *Sacculospora*. When the molecular taxa were considered, the genus of *Glomus* was identified with the most of OTUs with 232 and was the dominant genus in this area. The second dominant genus is *Septoglomus*, with 218 OTUs accounting for the total percentage of 31.1%. At the same time, only one OTU is identified in the genera of *Corymbiglomus* and *Paraglomus.*

When considering the AMF species and OTUs at different altitudes, both the numbers of species and OTUs in low-altitude areas were higher than those in high-altitude areas. The highest numbers of OTUs occurred at 1,800 m, and the lowest appeared at 3,100 m with 342 and 128, respectively.

### The Change of AMF Diversity Among Different Elevations

**FIGURE 2:**
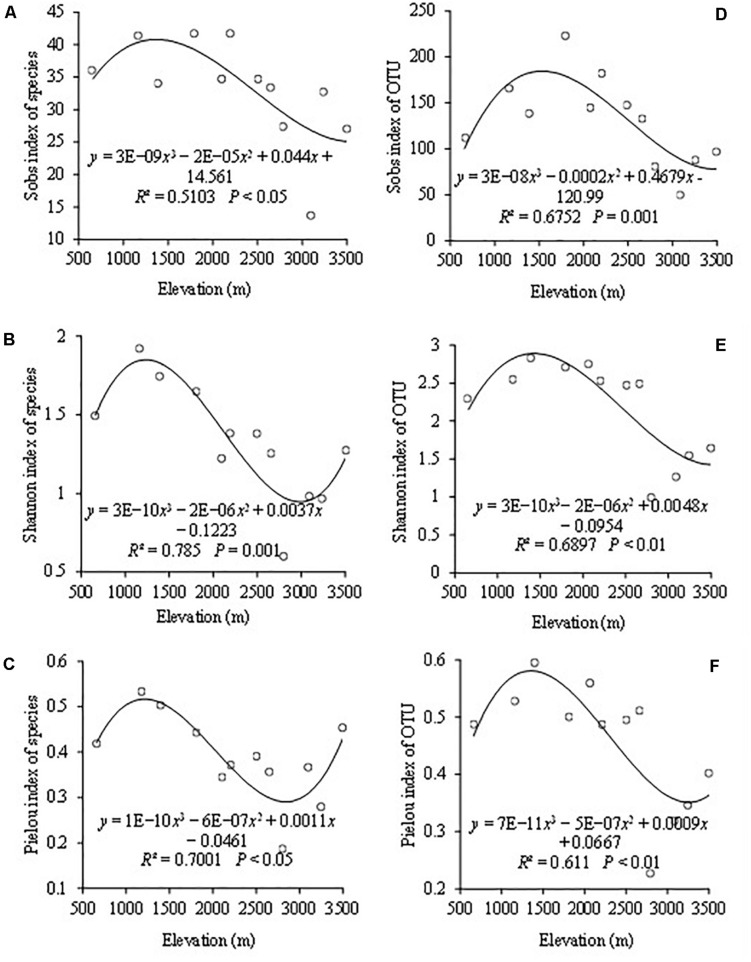
The variation of AMF based on the species and OTU level among different elevations with Sobs index of species **(A)**, Shannon–Wiener index of species **(B)**, Pielou index of species **(C)**, Sobs index of OTUs **(D)**, Shannon–Wiener index of OTUs **(E)**, and Pielou index of OTUs **(F)**.

The indices of Sobs, Shannon, and Pielou represented the AMF community richness, diversity, and evenness, respectively. When based on the species level, the highest species richness occurred at the elevations of 1,800 and 2,200 m (Figure 2A). The highest Shannon diversity and Pielou evenness both appeared at 1,170 m (Figures 2B,C). The lowest Shannon diversity and Pielou evenness of AMF were both observed at 2,850 m. Cubic functions could be used to simulate the change trends of Sobs index, Shannon index, and Pielou index with *R*^2^ values of 0.51, 0.78, and 0.70, respectively, as the altitude changes (Figures 2A–C).

When based on the OTU level, AMF OTU richness ranged from 49.7 to 223 with a mean of 130 (Figure 2D). The highest and lowest points of the OTU richness appeared at elevations of 1,800 and 3,100 m, respectively. The highest and lowest points of Shannon diversity and Pielou evenness both appeared at the same altitudes with the highest at 1,400 m and the lowest at 2,850 m (Figures 2E,F). The changing trends of the OTU diversity indices including Sobs, Shannon, and Pielou were consistent, and they could be simulated by a cubic function with the *R*^2^ values of 0.675, 0.690 and 0.611 (Figures 2D–F). Besides, altitude revealed a significant effect on them.

### Abundance and Occurrence Frequency of AMF Genera

Based on OTUs and species abundances, we obtained the relative abundance of genera at the species and OTU levels (Table 2). The fungi in the genus of *Glomus* are the most dominant, with the highest relative abundance based on species and OTUs. Besides, *Glomus* is found in all altitudes with the occurrence frequency of 100%. The second abundant genus is *Septoglomus* with the relative abundance of 8.247% and 31.054% at the species and OTUs levels. The occurrence frequency of *Septoglomus* is also higher with 97.22%. In addition, some genera have the same occurrence frequency with 88.889%, including *Acaulospora*, *Ambispora*, and *Rhizophagus*. Although the relative abundance of *Ambispora* is lower, the occurrence frequency is higher, with 88.889%. The fungi in the genera of *Corymbiglomus* and *Sacculospora* had the least relative abundance and occurrence frequency in Mt. Taibai. Besides, an unidentified genus is found with the occurrence frequency of 33.33%.

**TABLE 2:** Relative abundance and occurrence frequency of AMF genus in Mt. Taibai.

**Genus**	**Relative abundance on species level (%)**	**Relative abundance on OTU level (%)**	**Occurrence frequency (%)**
*Acaulospora*	12.371	11.538	88.889
*Ambispora*	1.031	1.994	88.889
*Claroideoglomus*	4.124	2.422	80.556
*Corymbiglomus*	1.031	0.142	2.778
*Diversispora*	5.155	2.422	38.889
*Redeckera*	1.031	0.285	13.889
*Entrophospora*	2.062	0.285	19.444
*Scutellospora*	2.062	0.427	36.111
*Funneliformis*	3.093	1.709	80.556
*Glomus*	42.268	33.048	100.000
*Kamienskia*	2.062	3.846	55.556
*Dominikia*	2.062	0.712	75.000
*Rhizophagus*	8.247	7.123	88.889
*Sclerocystis*	1.031	0.285	22.222
*Septoglomus*	8.247	31.054	97.222
*Pacispora*	1.031	0.712	25.000
*Paraglomus*	1.031	0.142	11.111
*Sacculospora*	1.031	0.285	30.556
*unidentified*	1.031	1.567	33.333

### Effects of Soil Factors on AMF Diversity

The concentrations of C and N ranged from 4.44 to 16.69 and 0.41 to 1.82 g ⋅ kg^–1^, respectively, in Mt. Taibai (Table 3). Besides, the highest and lowest concentrations of C and N both occurred at the same elevations at 1,800 and 2,100 m, respectively. The highest soil P concentration is 111.80 mg ⋅ kg^–1^ occurring at a.s.l. 2100 m, which significantly higher than other altitudes. The range of pH is 5.56 to 7.17, with the highest and lowest values at a.s.l. 2,100 and 2,850 m, respectively.

**TABLE 3:** The contents of soil factors at different altitudes.

**Elevations (m)**	**C (g ⋅ kg^–1^)**	**N (g ⋅ kg^–1^)**	**Available P (mg ⋅ kg^–1^)**	**pH**
660	7.00 ± 0.76*fgh*	0.70 ± 0.06*fhg*	59.97 ± 12.79*bc*	7.13 ± 0.52a
1,170	7.99 ± 0.33*efg*	0.81 ± 0.03*defg*	23.35 ± 1.66*de*	6.87 ± 0.35a
1,400	6.95 ± 1.06*fgh*	0.78 ± 0.10*efh*	32.95 ± 8.40*de*	6.64 ± 0.19*abc*
1,800	16.69 ± 1.71a	1.82 ± 0.14a	108.61 ± 7.10b	6.45 ± 0.05*abcd*
2,100	4.44 ± 0.78h	0.41 ± 0.07i	111.80 ± 7.82a	7.17 ± 0.04a
2,200	10.16 ± 1.06*de*	1.06 ± 0.11*bcd*	34.92 ± 5.40*de*	6.73 ± 0.11*ab*
2,500	9.65 ± 0.81*def*	0.95 ± 0.08*cdef*	77.88 ± 12.20b	6.43 ± 0.11*abcd*
2,650	5.51 ± 0.30*gh*	0.58 ± 0.03*ghi*	19.80 ± 2.70*de*	5.94 ± 0.14*bcd*
2,850	14.46 ± 0.52*ab*	1.23 ± 0.05b	28.66 ± 5.60*de*	5.56 ± 0.45d
3,100	13.58 ± 0.94*bc*	1.18 ± 0.07*bc*	39.97 ± 4.56*cd*	6.44 ± 0.01*abcd*
3,250	5.91 ± 0.31*gh*	0.51 ± 0.04	12.84 ± 0.90*e*	5.60 ± 0.41d
3,500	11.23 ± 1.45*cd*	1.05 ± 0.15*bcde*	26.40 ± 1.57*de*	5.81 ± 0.15*cd*

*Different lowercase letters in the same column indicate significant difference (*P* < 0.05).*

The results showed that AMF OTU richness and root colonization are not significantly correlated with soil factors (C, N, pH, and available P) (Table 4). The carbon significantly correlated with the indices of Shannon (*r* = −0.339, *P* < 0.05) and Pielou (*r* = −0.405, *P* < 0.05). Soil pH also has significant effects on the index of Shannon (*r* = 0.431, *P* < 0.01) and Pielou (*r* = 0.375, *P* < 0.05). However, the N and available P have no significant effect on AMF diversity.

**TABLE 4:** Influence of soil factors on AMF diversity.

**Soil factor**	**Shannon index**	**Sobs index**	**Pielou index**	**Colonization**
C	−0.339*	0.012	−0.405*	−0.090
N	–0.140	0.197	–0.239	−0.041
pH	0.431**	0.305	0.375*	−0.174
Available P	0.324	0.328	0.238	−0.200

*Correlation is significant at the 0.05 level.**Correlation is significant at the 0.01 level.

## Discussion

Mountain ecosystem has more complex structures and functions because of their pronounced climatic gradients within relatively short distances. Therefore, AMF community distribution and diversity are affected by many factors in mountain ecosystem, such as host plant types, vegetation coverage, environment factors, and so on (Bauer et al., 2020). Different elevation gradients provide a unique opportunity to explore AMF community distribution and diversity, thus laying the foundation for further exploration of the driving forces that lead to species aggregation (Wagg et al., 2011). Therefore, clarifying the response of AMF diversity and distribution to changes in elevations is indispensable to improve understanding of the function of microorganisms in mountain ecosystems (Yang G.W. et al., 2016).

In the current study, the highest of AM root colonization reached 87.7%, with an average 56.1%. It could be seen that AMF can form a good symbiosis with plants in the mountain. The root colonization was higher than the result of our study on Mt. Taibai in 2014. It is possible that the adaptability of the AMF to the local environment has increased. Studies have shown that the adaptation of microbial communities to local conditions may have a potential role in shaping the composition of plant communities (Bauer et al., 2020). Therefore, AMF’s adaptability to environmental conditions may enhance its symbiosis ability with plant roots. The variation trend of the cubic function of AMF colonization was roughly the same with 2014 with the change of altitudes. Besides, our results showed that altitude had a significant effect on AM colonization. This is inconsistent with the conclusion drawn by Li et al. (2020), who indicated that root colonization had no significant difference between the high and low altitudes in the Southeast of Tibetan Plateau. However, Kotilinek et al. (2017) suggested that root colonization decreased with the increase of altitude in Mt. Segrila. These different results may be due to the differences in plant species, geographic location, and hydrothermal environment. Besides, in this study, the plant species have a great difference at different altitudes in Mt. Taibai (Supplementary File 1), which makes the difference in the symbiotic relationship between AMF and roots (Jansa et al., 2008; Davison et al., 2011).

The 702 OTUs and 103 species of AMF were isolated and represented 18 identified and 1 unidentified genus in the soil sample based on sequence similarities. In previous investigations, morphological method identified 63 AMF species representing 12 genera formed in Taibai mountain soil samples in 2014. This result showed that the AMF was more abundant as identified by the molecular method than identified by the morphological method. And it further proves the conclusion that compared to molecular ecology methods, the level of AMF diversity is underestimated as identified by the morphological method, especially in natural ecosystems with little human interference (Ohsowski et al., 2014). A large number of AMF OTUs were identified in this study, which confirms the view that AMF has a wide ecological range and is an important part of mountain ecosystems (Liu et al., 2015). The members of OTUs in *Glomus*, *Kamienskia*, and *Septoglmus* genera prevailed at lower altitudes, and *Corymbiglomus* only appeared at a lower altitude. And the numbers of OTUs in *Funneliformis* and *Scutellospora* are higher at altitudes greater than 2,500 m than at low altitudes. The distribution of AMF communities at different altitudes also verifies the general assumption in microbial biogeography that “everything is everywhere, but the environment will choose” (Li and Ma, 2016). Overall, the numbers of species and OTUs were higher at low altitudes than high altitudes. The main reason may be that high-altitude organisms were more susceptible to environmental stress than low-altitude plants (Ahmad et al., 2020).

In addition, the result also corresponded to the habitat hypothesis that AMF communities follow changes in abiotic conditions.

*Glomus* is the dominant genus with the largest number of OTUs at each altitude. It was inconsistent with the previous conclusion that *Acaulospora* was the dominant genus identified by morphological methods in 2014. The reason for the different results was probably due to different identification methods. It also may be due to changes in soil nutrients and clime that lead to the reduction of *Acaulospora* and enhance the dominance of *Glomus*, so it shows that *Glomus* becomes more and more adapted to environmental changes over time (Camenzind et al., 2014). This result proved that as the main participant and regulator of the soil material cycle, AMF could sensitively sense and respond to small changes in the soil ecosystem and also showed that mycorrhizal symbiotes played a critical role in responding to climate change (Shi et al., 2017; Panneerselvam et al., 2020). Bonfim et al. (2016) study suggested that *Glomus* and *Acaulospora* were the dominant genera in a Brazilian Atlantic forest. Zhao et al. (2017) insisted that *Glomus* was predominated in all soil types: 60%–75% in grassland, 70%–75% in arable land, and 50%–70% in forest land. This was consistent with our result. In addition to the difference in relative abundance of each genus at the species and OTUs levels, the occurrence frequency of each genus was also different in this study. These differences might count for the change of AMF community distribution.

In addition to different AMF colonization and distribution, the diversity of AMF at species and OTUs levels was also different at different altitudes. On the whole, Sobs, Pielou, and Shannon indices all tended to a trend of cubic function. Besides, the highest species evenness and Shannon diversity both appeased at low altitude at 1,170 m and had a decreasing trend at mid-altitude. The reasons for this result may be that compared with high altitude and low altitude, the plant’s species replacement rate was less at mid-altitude (Ahmad et al., 2020). At the same time, they are also likely affected by large-scale climate and environment forcing. So, in this study, AMF diversity indices of Sob, Shannon diversity, and Pielou evenness had a decreasing trend at mid-altitude. The highest species richness of AMF was more than 40, which was far higher than the previous conclusion that the highest species richness was 18 in 2014. It seems to confirm this conclusion that molecular biology techniques can detect the species with higher similarity sequences (Chamkhi et al., 2018).

AMF OTU richness showed a changing trend of cubic function with the increase of the altitude. Some studies showed that AMF richness decreased with the increase in elevation (Lugo et al., 2008; Shi et al., 2019). In their research Yang W. et al. (2016) found that elevation did not significantly affect AMF OTUs richness. In addition, we found that the OTU richness at all altitudes from 49 to 223 was more abundant than those in previous studies (Liu et al., 2017; Shi et al., 2019). It can be seen that AMF richness was higher in Mt. Taibai. It may be because of the special geographical location and environment that make microorganism richness in Mt. Taibai higher than other mountains (Peng et al., 2018), or it may be because the mountain has higher plant species richness (Chi and Tang, 2011). Hiiesalu et al. (2014) found a positive relationship between plant richness and AMF richness. In addition to plant richness, which could influence the AMF richness, AMFs can also enhance the stability of plant community by mediating the compensatory effect between plant species and functional groups (Yang W. et al., 2016). O’Connor et al. (2002) also indicated that AMFs affect the relative abundance of different plant species by affecting the physiological and biochemical activities of plant species, thus changing plant community structure and species diversity.

Consistent with AMF richness, OTU’s Shannon diversity and Pielou evenness showed a cubic function trend with increasing altitude. And altitude had a significant impact on them. However, some studies suggested that AMF evenness was not a significant association with the changes of altitudes (Li et al., 2014; Shi et al., 2019). These different results could be caused by many factors. The mountain environment is inherently complex and changeable. In recent years, natural factors such as climate, environment, and human activities have caused changes in ecosystem service functions, which may cause the change of species. Besides, some studies have suggested that the host plant was a main driving factor of mycorrhizal fungal community distribution during the mountain ecosystem development (Li et al., 2014; Martinez-Garcia et al., 2015). Of course, the relationship between AMF and host plants needs to be further explored in this area.

Although the diversity indices showed the same changing trend with the increase of altitudes, the highest point of each diversity index appeared at different altitudes at the species and OTU levels. Besides, the average values of Sob, Shannon, and Pielou index at the OTU level were all higher than the species level. The reason for this result may be related to the difference in the relative abundance of each genus on the species and OTU levels. The relative abundance of each genus was also different at different altitudes (Supplementary File 3). Moreover, the occurrence frequency of each genus was also different, which may be also closely related to the above result. The change mechanism of the AMF diversity at species and OTU levels with altitudes still needs our further exploration. In general, the effect of altitude on species and OTU diversity may be the result of a combination of environmental factors, plant species, soil, and other factors.

Altitude gradient affects the community distribution of AMF mainly by affecting temperature and precipitation (Koske and Gemma, 1997). From the bottom to the top, the essence of the elevation rise is the continuous decline in temperature and precipitation, which leads to changes in soil factors. Along this elevation gradient, the corresponding changes in soil factors have affected the AMF community and diversity to varying degrees. Studies have shown that adding nutrients generally reduces the diversity and abundance of AMF (Carrara et al., 2018; Jeske et al., 2018). Consistently, C and N are negatively correlated with the Shannon diversity and Pielou evenness of AMF in this study. It may be due to changes in soil nutrients that lead to the reduction or loss of some fungal species and enhance the dominance of certain AMF species (Camenzind et al., 2014). Furthermore, the enrichment of C and N may interfere with the long-term adaptability of AMF to soil environment, thereby reducing the diversity and evenness of AMF (Cotton, 2018; Ma et al., 2020).

In the study of Ji and Bever (2016), soil available P played an important role in AMF community structure. However, Moora et al. (2014) pointed out that soil available P did not play a significant role in AMF community distribution, which is consistent with our study. It was also consistent with our study in 2014 that soil P showed a negligible effect on AMF community distribution. The reason may be that as a sedimentary mineral element, the concentration of P is relatively stable and has low mobility in the soil. Studies have shown that the roots of plants have two ways to absorb phosphorus from the soil. In P-rich soil, the roots directly absorb phosphorus (Smith et al., 2015; Raven et al., 2018). In P-limited soil, roots can undergo symbiosis with AMF and thus facilitate hyphae to absorb phosphorus (Johnson et al., 2015). Thus, there are studies that have shown that with the improvement of soil P utilization, the diversity and abundance of AMF decrease (De Beenhouwer et al., 2015; Ceulemans et al., 2019). Therefore, the change of soil available P concentration is an important predictor of the response of AMF diversity and abundance to soil nutrients. In this study, the pH was closely correlated with the Shannon diversity and Pielou evenness. Some studies have shown the same conclusion. For example, Xu et al. (2016) suggested that soil pH was the strongest predictor of AMF diversity and richness. Rozek et al. (2020) also showed that the relationship between soil pH and the indices of Shannon and Pielou evenness of AMF provides important information for predicting AMF community changes. The reason may be that soil pH directly affects the formation and germination of AMF spores, and compared with pH 5.5, more spores were found in soil with neutral pH (pH 6.5) (Jamiolkowska et al., 2018). And the changes of soil pH can directly impose physiological constraints on AMF. When soil pH exceeds a certain range, it may cause changes in AMF communities (Xu et al., 2017). Thus, soil pH may be a selective factor for the composition of rhizosphere microorganisms in the soil (Bucking and Kafle, 2015). Therefore, soil factors play an important role in AMF community and diversity.

This is the first study to explore AMF molecular diversity and species richness in Mt. Taibai. This study sampled from the altitudes of 660 to 3,500 m, which can reflect the AMF distribution and diversity of the whole mountain. However, because of the limitation of sampling time, the AMF distribution and diversity may be affected by the season and the degree of dryness and wetness (Guadarrama et al., 2014). Besides, the plant diversity hypothesis predicts that the higher the richness of plant species, the more microbial niches are available, making it more likely that microbes will find an eligible host (Waldrop et al., 2006). So, in the future, we are going to study the impact of different plant species on the AMF distribution and diversity at different elevations.

## Conclusion

There are abundant AMF molecular diversity and species richness in Mt. Taibai. AMF diversity measured by molecular method is much more than the previous findings by morphological methods. Of AMFs, 702 OTUs belonging to 103 species are isolated and identified representing 18 identified and 1 unidentified genus. The fungi in the genus of *Glomus* are the most dominant, which present in all elevations. AMF diversity indices showed a trend of cubic function on species and OTU level with the increase of altitudes. However, AMF diversity is higher in the middle to low-altitude than high-altitude areas. These findings suggested that elevation is the main factor governing the AMF diversity and distribution pattern.

## Data Availability Statement

The datasets presented in this study can be found in online repositories. The names of the repository/repositories and accession number(s) can be found below: https://www.ncbi.nlm.nih.gov/sra/PRJNA692777.

## Author Contributions

MZ and ZS performed conceptualization, resources, data curation, writing review and editing, and visualization. MZ performed methodology, software, formal analysis, and prepared original draft. MZ, ZS, and MY performed validation. MZ, ZS, MY, and SL performed investigation. ZS performed supervision, project administration, and funding acquisition. All authors contributed to the article and approved the submitted version.

## Conflict of Interest

The authors declare that the research was conducted in the absence of any commercial or financial relationships that could be construed as a potential conflict of interest.
