# Supplementary material for: Molecular Diversity and Distribution of Arbuscular Mycorrhizal Fungi at Different Elevations in Mt. Taibai of Qinling Mountain
Source: Front Microbiol. 2021 Mar 4;12:609386. doi: 10.3389/fmicb.2021.609386 (PMC7974767; doi:10.3389/fmicb.2021.609386)
Supplement: Supplementary file 1 [file Table_1.DOCX]

**Supplementary materials**

Supplementary File S1. Soil properties and vegetation of study sites in each elevation along altitudinal gradients of Mt. Taibai.

| Elevation (m) | Soil types | vegetation types | Main plant species |
| --- | --- | --- | --- |
| 660-1100 | Cinnamon soil | shrubs | *Acer grosseri, Agrimonia pilosa, Artemisia viridissima, Celastrus orbiculatus, Cercis chinensis, Cotinus coggygria, Diospyros lotus, Galium handelii, Lonicera ferdinandii, Lonicera hispida, Quercus aliena var. acuteserrata, Quercus variabilis, Rosa tsinglingensis, Thalictrum robustum* |
| 1200 | Cinnamon soil | forest | *Acer grosseri, Carex japonica, Carpinus cordata, Carpinus shensiensis, Cotinus coggygria, Litsea pungens, Paederia scadens, Quercus aliena var. acuteserrata, Quercus variabilis, Rosa tsinglingensis, Rubia cordifolia, Rubus sp., Smilax discotis, Spiraea blumei, Viburnum dilatatum* |
| 1350 | Cinnamon soil | forest | *Acanthopanax stenophyllus, Acer grosseri, Carpinus turczaninowii, Deyeuxia sinelatior, Litsea pungens, Quercus variabilis, Sanguisorba officinalis, Scutellaria honanensis, Spiraea pubescens, Toxicodendron verniciflum, Viola mandshurica* |
| 1500 | Brown soil | forest | *Acer grosseri, Agrimonia pilosa, Akebia trifoliata, Carex siderosticta, Celtis koraiensis, Lespedeza formosa, Litsea pungens, Litsea tsinlingensis, Lonicera japonica, Quercus aliena var. acuteserrata, Smilax scobinicaulis, Sorbus alnifolia, Spiraea mongolica, Toxicodendron verniciflum* |
| 1650 | Brown soil | forest | *Acer grosseri, Acer robustum, Acer shankanensis, Agrimonia pilosa, Carex siderosticta, Crataegus kansuensis, Festuca modesta, Lespedeza formosa, Litsea pungens, Lonicera japonica, Maddenia hypolenca, Quercus aliena var. Acuteserrata, Rubus sp.* |
| 1800 | Brown soil | forest | *Acer shankanensis, Berberis dolichobotrys, Carex japonica, Carpinus cordata, Cotoneaster divariacatus, Euonymus alatus, Euonymus phellomanus, Euonymus verucosoides, Lespedeza formosa, Lindera obtusiloba, Quercus liaotungensis, Rubia cordifolia, Sorbus alnifolia, Thalictrum robustum* |
| 1950 | Brown soil | forest | *Acanthopanax henryi, Acer henryi, Ampelopsis megalophylla, Carpinus cordata, Cornus controversa, Fraxinus inopinata, Lindera obtusiloba, Pyrus betulaefolia, Quercus liaotungensis, Rosa swiginzowii, Rubia cordifolia, Sorbaria arborea var. glabrata, Viola mandshurica, Viola sp.* |
| 2100 | Brown soil | forest | *Acer grosseri, Acer miaotaiensis, Carex japonica, Carpinus cordata, Litsea pungens, Lonicera serreana, Pinus armandii, Populus purdomii, Quercus liaotungensis, Rosa davurica, Rubia cordifolia, Spiraea blumei, Viburnum dilatatum* |
| 2250 | Brown soil | forest | *Abelia engleriana, Betula albosinensis, Crataegus hupehensis, Deyeuxia sinelatior, Euphorbia pekinensis, Fraxinus mandschurica, Lonicera ferdinandii, Pinus armandii, Quercus liaotungensis, Rosa swiginzowii, Salix characta, Sorbaria arborea var. Glabrata, Spiraea mongolica, Viola pekinensis* |
| 2400 | Brown soil | forest | *Abelia engleriana, Acer henryi, Betula albosinensis, Corylus ferox, Corylus heterophylla var. Sutchuenensis, Deyeuxia sinelatior, Euonymus venosus, Euphorbia pekinensis, Fraxinus inopinata, Fraxinus mandschurica, Lonicera ferdinandii, Lonicera serreana, Quercus liaotungensis, Viola pekinensis* |
| 2550 | Brown soil | forest | *Acer giraldii, Betula albosinensis, Carex japonica, Hydrangea bretschneideri, Lonicera ferdinandii, Lonicera japonica, Lonicera serreana, Lonicera tangutica, Phlomis umbrosa var. Stenocalyx, Pinus armandii, Quercus liaotungensis, Rosa omeiensis, Rubia membranacea, Rubus sp., Sorbus koehneana* |
| 2700 | Brown soil | forest | *Acer giraldii, Acer mono var. Tricuspis, Betula albo-sinensis var. Septentrionalis, Carex japonica, Euonymus alatus, Fragaria corymbosa, Oxylis corniculata, Rhododendron purdomii, Rosa omeiensis, Rubia cordifolia, Salix paraplesia, Sorbaria arborea var. Glabrata, Sorbus folgneri* |
| 2850 | Dark brown soil | forest | *Abelia engleriana, Abies fargesii, Acer shankanensis, Berberis dolichobotrys, Berberis feddeana, Betula albo-sinensis, Betula albo-sinensis var. Septentrionalis, Carex japonica, Lonicera webbiana, Lonicera webbiana, Pinus armandii, Potentila glabra, Rhododendron purdomii, Rubia cordifolia, Sorbus folgneri, Spiraea pubescens* |
| 3000 | Dark brown soil | forest | *Abies fargesii, Betula albo-sinensis var. Septentrionalis, Callicarpa japonica var.angustata, Cardamine macrophylla, Carex japonica, Fragaria gracilis, Larix chinensis, Ligularia dentata, Lonicera serreana, Oxylis corniculata, Potentila glabra, Rosa tsinglingensis, Rubus amabilis, Spiraea mongolica, Viola bifloara* |
| 3150 | Mountain meadow soil | forest | *Abies chensiensis, Acanthopanax giraldii, Berberis cicumserrata, Cardamine macrophylla, Carex japonica, Chrysosplenium pilosum var. Valdepilosum, Festuca modesta, Fragaria gracilis,* *Larix chinensis, Lonicera serreana, Oxylis corniculata, Rhododendron rufun, Rubus sp.* |
| 3300 | Mountain meadow soil | forest | *Abies chensiensis, Abies fargesii, Anemone taibaiensis, Berberis cicumserrata, Carex scabrirostris, Festuca modesta, Larix chinensis, Lonicera taipeiensis, Lonicera webbiana, Oxylis corniculata, Potentila glabra, Rhododendron rufun, Saxifraga montana* |
| 3450 | Alpine meadow | Shrub and grassland | *Adenophora stricta, Anaphalis hancockii, Cardamine macrophylla, Carex japonica, Carex scabrirostris, Epilobium palustre, Festuca modesta, Parnassia delavayi, Polygonum sphaerostachyum, Potentila glabra, Rhododendron capitatum, Salix paraplesia, Saxifraga montana, Spiraea alpina, Viola bifloara* |
| 3600 | Alpine meadow soil | Shrub and grassland | *Cardamine macrophylla, Carex japonica, Carex scabrirostris, Festuca modesta, Pedicularis giraldiana, Polygonum sphaerostachyum, Potentila glabra, Rhododendron capitatum, Salix characta, Saussurea iodostegia, Saussurea licentiana, Saxifraga montana* |
