# Supplementary material for: Molecular Diversity and Distribution of Arbuscular Mycorrhizal Fungi at Different Elevations in Mt. Taibai of Qinling Mountain
Source: Front Microbiol. 2021 Mar 4;12:609386. doi: 10.3389/fmicb.2021.609386 (PMC7974767; doi:10.3389/fmicb.2021.609386)
Supplement: Supplementary file 2 [file Table_2.DOCX]

Supplementary File S2. AMF species.

| Genus | Species |
| --- | --- |
| *Acaulospora* | Acaulospora alpine, Acaulospora baetica, Acaulospora cavernata, Acaulospora herrerae, Acaulospora brasiliensis, Acaulospora nivalis, |
|  | Acaulospora punctata, Acaulospora pustulata, Acaulospora viridis |
|  | Acaulospora scrobiculata, Acaulospora spinosa, Acaulospora sp. As2,  Acaulospora sp. As7 |
| *unidentified* | Acaulosporaceae sp. |
| *Ambispora* | Ambispora gerdemannii |
| *Claroideoglomus* | Claroideoglomus claroideum, Claroideoglomus drummondii, |
|  | Claroideoglomus etunicatum, Claroideoglomus luteum |
| *Corymbiglomus* | Corymbiglomus corymbiforme |
| *Diversispora* | Diversispora arenaria, Diversispora insculpta, Diversispora sp. W5257 |
|  | Diversispora varaderana, Diversispora trimurales |
| *Dominikia* | Dominikia achra, Dominikia aurea, Dominikia bernensis |
| *Entrophospora* | Entrophospora infrequens, Entrophospora sp. CL283 |
| *Funneliformis* | Funneliformis caledonium, Funneliformis mosseae  Funneliformis geosporum |
| *Glomus* | Glomus aggregatum, Glomus cf. claroideum, Glomus macrocarpum, |
|  | Glomus sp. 1, Glomus sp. 11, Glomus sp. 12, Glomus sp. 16,  Glomus sp. 18, Glomus sp. 22, Glomus sp. 23, Glomus sp. 25,  Glomus sp. 26, Glomus sp. 3, Glomus sp. 6, Glomus sp. 7,  Glomus sp. 8, Glomus sp. 9, Glomus sp. Att1485-12, Glomus sp. hr43,  Glomus sp. BEG104, Glomus sp. DQF04, Glomus sp. DS, |
|  | Glomus sp. HM-CL4, Glomus sp. hr16, Glomus sp.WFVAM23,  Glomus sp. hr73, Glomus sp. hr74, Glomus sp. Rp10, |
|  | Glomus sp. rp20, Glomus sp. rp25, Glomus sp. rp29, Glomus sp. rp40, Glomus sp. rp38, Glomus sp. rp12, Glomus sp. rp65, Glomus sp. SR |
|  | Glomus tetrastratosum, Glomus sp. hr11, Glomus sp. S326  Glomus sp. DQF05, Glomus sp. DQF06, Glomus sp. DQF07,  Glomus sp. DQF17, Glomus sp. UY110 |
| *Kamienskia* | Kamienskia bistrata, Kamienskia perpusilla |
| *Pacispora* | Pacispora scintillans |
| *Paraglomus* | Paraglomus occultum |
| *Redeckera* | Redeckera megalocarpum |
| *Rhizophagus* | Rhizophagus cf. intraradices, Rhizophagus cf. irregularis GCFI1, |
|  | Rhizophagus intraradices, Rhizophagus cf. irregularis MUCL 43205, |
|  | Rhizophagus irregularis, Rhizophagus invermaius,  Rhizophagus melanus, Rhizophagus sp. MUCL 43203,  Rhizophagus sp. MUCL46100 |
| *Sacculospora* | Sacculospora baltica |
| *Sclerocystis* | Sclerocystis sinuosa, |
| *Scutellospora* | Scutellospora calospora, Scutellospora sp. S247, |
| *Septoglomus* | Septoglomus africanum, Septoglomus altomontanum, |
|  | Septoglomus constrictum, Septoglomus jasnowskae,  Septoglomus viscosum, Septoglomus xanthium, Septoglomus fuscum, |
|  | Septoglomus sp. ZS-2014 |
