# Supplementary material for: Molecular Diversity and Distribution of Arbuscular Mycorrhizal Fungi at Different Elevations in Mt. Taibai of Qinling Mountain
Source: Front Microbiol. 2021 Mar 4;12:609386. doi: 10.3389/fmicb.2021.609386 (PMC7974767; doi:10.3389/fmicb.2021.609386)
Supplement: Supplementary file 3 [file Table_3.DOCX]

Supplementary File S3. Relative abundance on species level of AMF genus at different altitudes in Mt. Taibai

| Genus | Altitude (m) | | | | | | | | | | | |
| --- | --- | --- | --- | --- | --- | --- | --- | --- | --- | --- | --- | --- |
|  | 660 | 1170 | 1400 | 1800 | 2100 | 2200 | 2500 | 2650 | 2850 | 3100 | 3250 | 3500 |
| *Acaulospora* | 0.234 | 0.626 | 0.004 | 0.014 | 0.354 | 0.032 | 5.885 | 12.167 | 26.889 | 37.015 | 52.616 | 1.209 |
| *Ambispora* | 0.010 | 0.229 | 0.494 | 0.618 | 2.699 | 0.001 | 0.902 | 1.010 | 32.679 | 0.003 | 12.250 | 1.213 |
| *Claroideoglomus* | 0.112 | 1.924 | 15.018 | 0.496 | 4.146 | 0.009 | 0.025 | 0.486 | 0.008 | 10.844 | 0.004 | 0.006 |
| *Corymbiglomus* | 0 | 0.002 | 0 | 0 | 0 | 0 | 0 | 0 | 0 | 0 | 0 | 0 |
| *Diversispora* | 0.588 | 0.002 | 0.002 | 0 | 0.253 | 0.001 | 0.127 | 0.003 | 0.001 | 4.410 | 0 | 0.003 |
| *Dominikia* | 0.008 | 0.015 | 0.002 | 0.007 | 0.010 | 0 | 0.006 | 1.597 | 0.015 | 0.021 | 0.011 | 7.353 |
| *Entrophospora* | 0 | 0.033 | 0.002 | 0 | 0.099 | 0.003 | 0 | 0 | 0 | 0 | 0 | 0 |
| *Funneliformis* | 5.807 | 0.021 | 0.683 | 0.001 | 0.015 | 0.010 | 0.399 | 7.910 | 0.114 | 0.013 | 0.605 | 0.175 |
| *Glomus* | 27.493 | 43.018 | 23.497 | 46.232 | 8.898 | 0.128 | 14.938 | 10.436 | 0.288 | 41.628 | 1.096 | 24.721 |
| *Kamienskia* | 0.504 | 31.195 | 9.456 | 0.221 | 0.039 | 0.002 | 0 | 0 | 0 | 0 | 0.031 | 0.004 |
| *Pacispora* | 0.003 | 0 | 0 | 0 | 0 | 0 | 0.078 | 0 | 0 | 4.544 | 0.001 | 0.001 |
| *Paraglomus* | 0 | 0 | 0 | 0 | 0.007 | 0 | 0 | 0 | 0.001 | 0 | 0.004 | 0 |
| *Redeckera* | 0 | 0 | 0 | 0 | 0.031 | 0 | 0.017 | 0 | 0 | 0.049 | 0 | 0 |
| *Rhizophagus* | 0.445 | 1.246 | 1.214 | 10.432 | 6.537 | 0.177 | 0.003 | 0.120 | 0.004 | 0.005 | 0.125 | 43.505 |
| *Sacculospora* | 0.001 | 0 | 0 | 0 | 0.068 | 0 | 8.193 | 0.020 | 0.004 | 0.036 | 0 | 0 |
| *Sclerocystis* | 5.141 | 0 | 0 | 0 | 0 | 0 | 0.020 | 0 | 0 | 0 | 0.004 | 0.030 |
| *Scutellospora* | 0.003 | 0.014 | 0.049 | 0 | 0 | 0 | 0.217 | 0.066 | 0.014 | 0.005 | 2.575 | 0.001 |
| *Septoglomus* | 59.650 | 21.676 | 49.578 | 41.979 | 76.842 | 0.636 | 69.167 | 66.044 | 23.286 | 1.428 | 30.566 | 21.779 |
| *unidentified* | 0 | 0 | 0 | 0 | 0 | 0 | 0.024 | 0.141 | 16.687 | 0 | 0.112 | 0 |
