# Supplementary material for: Molecular Diversity and Distribution of Arbuscular Mycorrhizal Fungi at Different Elevations in Mt. Taibai of Qinling Mountain
Source: Front Microbiol. 2021 Mar 4;12:609386. doi: 10.3389/fmicb.2021.609386 (PMC7974767; doi:10.3389/fmicb.2021.609386)
Supplement: Supplementary file 4 [file Table_4.DOCX]

Table S4. Sample sequences

| #SampleID | Raw tags | Clean tags |
| --- | --- | --- |
| TB111 | 68731 | 57590 |
| TB112 | 100292 | 73780 |
| TB113 | 34478 | 33248 |
| TB131 | 94813 | 42005 |
| TB132 | 42305 | 37514 |
| TB133 | 103195 | 98668 |
| TB141 | 31276 | 30199 |
| TB142 | 35872 | 33883 |
| TB143 | 51854 | 50020 |
| TB151 | 28916 | 27713 |
| TB152 | 36728 | 35680 |
| TB153 | 48628 | 47254 |
| TB162 | 31694 | 30698 |
| TB163 | 43686 | 38009 |
| TB164 | 31549 | 30570 |
| TB181 | 69288 | 64148 |
| TB183 | 35851 | 34528 |
| TB184 | 31823 | 30370 |
| TB211 | 29464 | 28578 |
| TB213 | 85742 | 82069 |
| TB214 | 69892 | 67942 |
| TB21 | 91460 | 86230 |
| TB22 | 200960 | 193711 |
| TB23 | 91020 | 86677 |
| TB31 | 42231 | 39392 |
| TB32 | 99196 | 93934 |
| TB33 | 109983 | 105532 |
| TB41 | 92931 | 79775 |
| TB43 | 102663 | 99529 |
| TB44 | 102152 | 97256 |
| TB61 | 49245 | 48191 |
| TB62 | 62518 | 60291 |
| TB63 | 53651 | 51781 |
| TB81 | 50293 | 48898 |
| TB82 | 48632 | 46959 |
| TB83 | 56713 | 54369 |
